# Intra-uterine Tamponing

**Published:** 1888-06

**Authors:** 


### *INTRA-UTERINE TAMPONING.*

The subject of tamponing the uterus has just been revived by Doctor Duhrssen, who has obtained excellent results. Auvard, who successfully employed the method in a bad case of hemorrhage, inclines to the belief that it will prove valuable in puerperal septichæmia. He states as the two principal indications for use of the tampon:

1. Severe *post-partum* hemorrhage.
2. Septichæmia, arising from curetting or other operation interior of uterus.

You perform the operation as follows: Cleanse antiseptically vulva and vagina, and then pull the uterus towards you. In case of *post-partum* hemorrhage, it is unnecessary to move the uterus, as the hand can be passed directly into its cavity. Free the uterus of any clots or other material, if it be a case of septichæmia. Take a piece of iodoform gauze, about fifteen feet in length, six inches in width, and sufficiently thick, and insert this either with finger or hand, according to circumstances, into the uterus. Leave an end of the gauze in vagina. Compress the abdomen with a binder or corsets. After twenty-four hours, the tampon is removed by traction on the piece of gauze at the vulva.—*Lyon Medical.*

---

Incubation and Gavage.—At the February meeting of the Obstetrical Society of Philadelphia, Dr. Hirst exhibited the incubator in use at the Maternity Hospital. It is a simplified Crede's incubator, a double-walled bath-tub made of copper; hot water is poured into the space between the walls, and a temperature maintained within the tub of nearly 100° Fahr. He also described, in all its details, the system of gavage in use at the same hospital. It consists in forcing into the child's stomach, through a soft rubber catheter, by means of a small glass syringe, about 1½ drachms of human milk every hour. A table showing the daily weight of a premature infant, born at the twentieth day and treated by this method, was presented. The child weighed, at first, 1080 grammes; at the end of the first month, the weight was 1460 grammes. Tarnier, instead of a syringe, uses a glass in this treatment.—*Col. and Clin. Record, March,* 1888.

---

Statistics of Death from Poison.—There were in Great Britain, in 1886, 511 deaths from poison, including cases of chronic poisoning by lead. Of these, 327 were accidental, 178 suicidal, and only six homicidal. Lead heads the list of agents giving rise to accidental poisoning (95 cases), then follows opium and its derivatives (82 cases); carbolic acid (20 cases); belladonna is responsible for 9 cases; alcohol for 7; aconite, chlorodyne, and hydrochloric acid, each for 6; prussic acid, ammonia, and strychnine, each for 5. Carbolic acid was selected by 42 suicides; opium, laudanum or morphine by 41; oxalic acid by 28; prussic acid by 25; vermin-killer by 18; hydrochloric acid by 15; strychnine by 14. Sulphuric acid and arsenic have lost their popularity, the former having been used by only six and the latter by five persons. One is said (in the official report) to have died from "bichlorate" of potash, another from "bisulphate" of carbon and three from "mercury," probably in accordance with the verdicts of coroners' juries.

---

Eczema of the Nasal Orifice.—In the *Archiv. für Kinderheilk.*, Bd. ix., Herzog says that, in the greater number of cases, the eczema is combined with scrofula. In all cases, he has found chronic rhinitis coexistent. The affection is sometimes complicated by erysipelas or furunculosis, and he relates six cases of this complication. In adults, the affection is sometimes not easily differentiated from sycosis. The treatment of the author consists in the applications of ointments, of yellow oxide of mercury and vaseline with lead.

What Medical Men said of Anæsthetics Forty Years Ago.—Commenting on the reports of the first use of ether as an anæsthetic in surgery, the Philadelphia *Medical Examiner* expressed the views of the conservatives in the following terms: "We are persuaded that the surgeons of Philadelphia will not be seduced from the high professional path of duty into the quagmire of quackery by this will-o'-the-wisp. * * * We cannot close these remarks without again expressing our deep mortification and regret that the eminent men who have so long adorned the profession in Boston, should have consented for a moment to set so bad an example to their younger brothers, as we conceive them to have done in this instance. If such things are to be sanctioned by the profession, there is little need of reform conventions, or any other efforts, to elevate the professional character; physicians and quacks will soon constitute one fraternity."

---

Uterus: Indications for Washing Out.—According to a writer in *El Siglo Medico*, the following are the indications for the use of the intra-uterine antiseptic douche:

1. Fetid lochia with elevation of temperature.
2. Incomplete expulsion of the placenta.
3. Retention of membranes.
4. Birth of a putrid fœtus.
5. Slow involution of the uterus, due to the retention of clots.
6. Septichæmia coming on late in the puerperal period.
7. Cases of flexion of the uterus, which prevent the escape of the lochia.
8. When, after abortion, it is found necessary to curette the uterus.
9. Whenever it has been necessary to introduce the hand into the uterus.

---

Is there a Bacillus of Carcinoma?—Senger has made two hundred bacteriological experiments with ten pieces of various kinds of carcinoma. He has utilized all the known means of culture, following scrupulously Scheurlen's process.

The results have been negative, and Senger's conclusions are as follows: We cannot, by means of all the genuine methods, succeed in cultivating in fragments of cancer a bacterium or a coccus which has any connection whatever, from an etiological point of view, with carcinoma. It is not necessary, in order to trace bacteria, to believe that carelessness has introduced them into tumors; it is known that microbes introduce themselves into the galactophoric canals, and may thus gain entrance to the organism. Senger claims that the isolated bacillus cultivated and described by Scheurlen is only a bacillus of the potato, completely inoffensive. The bacilli of the potato, which, up to the present time, have not been carefully studied, are often found accidentally, and are not at all pathogenic. These bacilli grow upon potatoes by a pellicle which is waved and wrinkled; they dissolve gelatine and form on it also a pellicle. Scheurlen has certainly been misled by this inoffensive microbe.—*Le Progrès Medical.*

---

Substitutes for Digitalis.—Professor Luciani finds, after a careful study of the various agents that have been introduced as substitutes for digitalis, that strophanthus and adonis vernalis have nearly the same action on the heart that digitalis has, and are to be preferred, because they have no cumulative action and no injurious influence over digestion. Caffeine does not answer the purpose so well, and is apt to disturb digestion. Lily of the valley is greatly inferior in activity, although, in some mild cases, it may be useful. Sparteine is of no practical value, having little influence over irregular heart action, and producing, moreover, headache and other disagreeable effects.

---

The Treatment of Whooping Cough by Intral-Nasal Insufflations of Antiseptic Powders.—(Barbot: These, Paris, 1888.)—This is a thesis inspired by Dr. Moigard, who is making general, in France, the treatment of whooping cough by nasal insufflations, and in which the author makes a very interesting biographical study of the question. He says the treatment shortens the duration of whooping cough, diminishes the number and violence of the coughing attacks, and causes the vomiting and hemorrhage to cease. It is an important work.—*Journal of Laryngology*.

---

Formula for Salol.—The *Bul. Gen. de Therapeutique* contains the following formula for salol:

1. For burns, as a liniment application:

| | | PARTS. |
|---|---|---|
| ℞ | Ol. Olivæ | 60 |
| | Salol | 10 |
| | Siq. Calcis | 60 |

M. S. Liniment.

2. With collodium, for fissured nipples:

| | | PARTS. |
|---|---|---|
| ℞ | Salol | 4 |
| | Solve in— | |
| | Aetheris | 4 |
| | Adde— | |
| | Collodii Elastici | 30 |

3. Salol cotton is prepared by dipping cotton in a solution of equal parts of salol and ether. The ether evaporates and the salol remains equally distributed in the cotton.

---

An Inhalation for Phthisis.—In the *Rev. du Therapeutique* for December 1, 1887, Filleau and Petit give the following formula for inhalation in phthisis:

| | | |
|---|---|---|
| ℞ | Carbolic Acid | gr. 30 |
| | Essent. Terebinth | ʒ 12½ |
| | Essent. Picis. | ʒ 5 |
| | Eucalyptol | ʒ 7½ |
| | Chloroform | gtt. 5 |

M. S.—To be inhaled four to six times daily, for five minutes at each sitting.

CHRONIC GONORRHŒA.—Unna:

| | | PARTS. |
|---|---|---|
| ℞ | Ol Theobromatie | 100 |
| | Ceræ Flavæ | 5 |
| | Argenti Nitratis | 1 |
| | Balsami Peruviani | 2 |

M. S. Introduce a sound smeared with this preparation. If no benefit after three or four applications, repeat treatment after several days.

---

SCABIES.—Kaposi:

| | | PARTS. |
|---|---|---|
| ℞ | Naphthol | 15 |
| | Sapo Viridis | 50 |
| | Cretæ Præparatæ | 10 |
| | Adipis | 100 |

M. S. Apply but once, after which powder with starch.

---

GERANIUM MACULATUM IN CHRONIC BRONCHITIS.—The *Journal de Medecine*, of March 11, 1888, gives the following as a useful prescription in chronic bronchitis and bronchorrhœa:

| | | |
|---|---|---|
| ℞ | Tinct. Nucis. Vom. | ʒ 1. |
| | Tinct. sanguinariæ | ʒ 1. |
| | Ext. fluid. geranii. | ʒ 1½. |
| | Syrup. simpl. | ʒ 11¼. |

Of this, a drachm should be taken every four hours.—*Medical News, April* 14, 1888.

---

THE TREATMENT OF CONVULSIONS IN CHILDREN.—Simon has found the following, by rectal injection, most useful:

| | | |
|---|---|---|
| ℞ | Chloral hydrat. | gr. 15 |
| | Tinct. mosch. | gtt. 20 |
| | Aquæ | ʒ12½ to 15 |

This may be given in two rectal injections, care being exercised to avoid violence, which may irritate or injure the bowel. —*Gazette Medicale, March* 3, 1888.

PHENACETINE.—Dr. Robert Bell, writing to the *British Medical Journal*, says: "Will you permit me to call the attention of the profession to a new antipyretic and antineuralgic agent called 'phenacetine,' though, correctly speaking, it bears the chemical name of para-acetphenetidin?

"It can be given with perfect safety, in doses of from eight to twelve grains, every four hours. Phenacetine is a febrifuge which compares favorably with any of the modern antipyretics in the certainty of its effects and freedom from evil consequences. In fevers, its administration is followed by relief of the symptoms, and in sciatica, in which I have employed it very frequently, its utility cannot be overrated.

"Phenacetine is a white, crystalline powder, soluble with difficulty in water, but easily so in hot alcohol."

---

DYSMENORRHŒA.—Calvin:

℞ Tr. Gelsemii . . . . . . . . . . . . .

Tr. Camphoræ . . . . . . . . . . . .

Tr. Opii Camphoratæ . . . . . . . . . āā ʒ ij

M. S. Thirty drops every two hours p. r. n.

---

DYSMENORRHŒA.—Bartholow:

℞ Ex. Stramonii . . . . . . . . . . . .
Ex. Hyoscyami . . . . . . . . . . .
Ex. Opii . . . . . . . . . . . . . . . āā gr. vj.
M. et f. pilulas No. vj.
S. A pill every three, four or six hours.

---

ERYSIPELAS.—Fothergill:

℞ Plumbi Acetatis . . . . . . . . . . . gr. x.

Tr. Opii . . . . . . . . . . . . . . . ʒ iv.

Aquæ . . . . . . . . . . . . . . . ℥ xx.

M. S. Apply in charpie and cover with oiled silk.

---

MAGNESIUM SALICYLATE.—This salt, which is recommended by Huchard in the treatment of typhoid fever, is easily prepared by dissolving salicylic acid in boiling distilled water and saturating the hot solution with magnesium carbonate. It crystallizes in colorless needles, readily soluble in water and alcohol.

Peroxide of Hydrogen, according to Dr. Love, of St. Louis, is a most valuable agent in the treatment of diphtheria, ozæna, and in all cases of cancerous ulceration and of suppuration or necrosis. He employs it in a solution containing 0.5 to 3 per cent., using most frequently, however, a strength of one per cent., diluting the commercial "ten volume" peroxide with two or three times its volume of water. Of its value in clearing away and effectually deodorizing the decomposing exudate in cases of diphtheria, he speaks in the most emphatic terms, and he regards the remedy also as one of great usefulness in scarlet fever, whooping cough, and other specific diseases.

---

Antipyrine in Obstetrics.—In twenty cases, Queirel has used antipyrine; in fifteen of them with good results. Four grains can be injected subcutaneously, and repeated if there is no relief. Its action is felt in from twenty to twenty-five minutes. It is particularly valuable during dilatation of os. The woman scarcely feels the pains, and the contractions continue with regularity until complete dilatation.—*Journal de Medecine de Paris.*

---

Marson's Test for Sugar in the Urine.—Dissolve two grains of ferrous sulphate in about 150 minims of the urine, add five grains of caustic potassa, and boil. A dark green precipitate forms if sugar is present, and the supernatant liquid is reddish brown or black, according to the amount of sugar. When sugar is absent, the precipitate is greenish brown in color, and the liquid is colorless.—*London Medical Recorder, Feb. 20th.*

---

Glycerine in Enemata.—Fifty drops of glycerine, injected into the rectum, is a very efficient remedy for producing energetic and copious dejections. The action is dependent upon the property of the glycerine attracting water. There is a transfusion of water from the intestinal walls into the canal, followed by an afflux of blood to the parts and consequent desire to defecate.—*Medical Record.*

---

It may not be generally known among physicians that the bromide of lithium is almost a specific for muscular rheumatism. —*Bartholow.*

